# ensembldb: an R package to create and use Ensembl-based annotation resources

**DOI:** 10.1093/bioinformatics/btz031

**Published:** 2019-01-25

**Authors:** Johannes Rainer, Laurent Gatto, Christian X Weichenberger

**Affiliations:** 1 Institute for Biomedicine, Eurac Research, Affiliated Institute of the University of Lübeck, Bolzano, Italy; 2 de Duve Institute, UCLouvain, Brussels, Belgium

## Abstract

**Summary:**

Bioinformatics research frequently involves handling gene-centric data such as exons, transcripts, proteins and their positions relative to a reference coordinate system. The *ensembldb* Bioconductor package retrieves and stores Ensembl-based genetic annotations and positional information, and furthermore offers identifier conversion and coordinates mappings for gene-associated data. In support of reproducible research, data are tied to Ensembl releases and are kept separately from the software. Premade data packages are available for a variety of genomes and Ensembl releases. Three examples demonstrate typical use cases of this software.

**Availability and implementation:**

*ensembldb* is part of Bioconductor (https://bioconductor.org/packages/ensembldb).

**Supplementary information:**

Supplementary data are available at *Bioinformatics* online.

## 1 Introduction

When the human genome was released as a first draft (Lander *et al.*, 2001), researchers started to manage these kinds of large, fragmented and rapidly evolving complex datasets by creating genome browsing and database systems such as EMBL-EBI Ensembl (Birney *et al.*, 2004). The availability of a reference genome allows definition of a coordinate system, in which genomic data, such as collaboratively defined gene models, are described unambiguously by chromosomal positions. Ensembl publishes several data releases per year, rendering it a valuable resource for consistent and tightly integrated data. These data are used in high-throughput genomic data analyses, which are frequently carried out in the R statistical programing language using tools provided by Bioconductor (Huber *et al.*, 2015).

Here we present *ensembldb*, a Bioconductor package enabling the creation and usage of comprehensive, locally stored Ensembl-based offline annotation databases. In addition to gene model annotations we include protein annotations in the pre-built databases, offer a fast and powerful filter mechanism and provide functions for the mapping of arbitrary positions between the genome, exome, transcriptome and proteome.

## 2 Implementation and available data

Ensembl is one of the main annotation resources for genomic data with a web service for online data access and APIs enabling programmatic data access. The *ensembldb* package provides functions to retrieve annotations for any of the >300 species available through Ensembl and EnsemblGenomes using their Perl API and to store information in small custom databases, which can be distributed as self-contained SQLite files or MySQL databases. The annotations included in our EnsDb databases comprise (i) genomic coordinates for all genes, transcripts and exons of a species and their relation to each other; (ii) general metadata information such as gene and transcript biotypes, NCBI Entrez gene IDs; and (iii) protein annotations including amino acid sequences, positions of protein domains within these (from e.g. Pfam; Finn *et al.*, 2016) and mappings of Ensembl protein identifiers to UniProt accession numbers. Some of these annotations are also available in other Bioconductor annotation resources, in particular TxDb databases from the *GenomicFeatures* package (Lawrence *et al.*, 2013) providing genomic coordinates, or org*db packages that contain gene-related annotations. With *ensembldb*, all this information is bundled conveniently into a single database. We distribute pre-built EnsDb databases covering all Ensembl core species for a range of Ensembl releases using Bioconductor’s *AnnotationHub* resource, which can be thought of as a queryable repository for annotation data. These locally stored databases enable offline access to Ensembl annotations in Bioconductor, in contrast to the *biomaRt* package (Durinck *et al.*, 2009) that, while also providing Ensembl annotations, requires active internet connectivity.

In Bioconductor, the *AnnotationDbi* package provides a common interface for retrieving annotation data. Furthermore, the *GenomicFeatures* package defines means for representation, organization and structured retrieval of transcript models and genomic positions of genes and their exons. The *ensembldb* package is compliant with both interfaces, such that data retrieval and data access is handled in a standardized way.

In addition, we developed a powerful filtering framework in *ensembldb*, which directly translates to SQL queries for performance increase (benchmarks provided in the supplement). It is based on our AnnotationFilter classes, available as a separate Bioconductor package to encourage usage beyond *ensembldb*. This filtering framework can be classified into two main groups: one to query arbitrary textual information, such as gene symbols or UniProt accession numbers, and the other to handle positional information of genes, exons, transcripts and protein domains. Filters can be combined with logical expressions to create tailored queries and retrieve only specific data from the databases. This is particularly useful for visualizing transcript models from certain genomic regions: *ensembldb* facilitates plotting with Bioconductor packages *ggbio* (Yin *et al.*, 2012) and *Gviz* (Hahne and Ivanek, 2016).

Generally, results returned by *ensembldb* are compatible with the standards defined by Bioconductor, such that data can be easily exchanged with other packages for further analysis.

## 3 Usage and examples

**Fig. 1.**
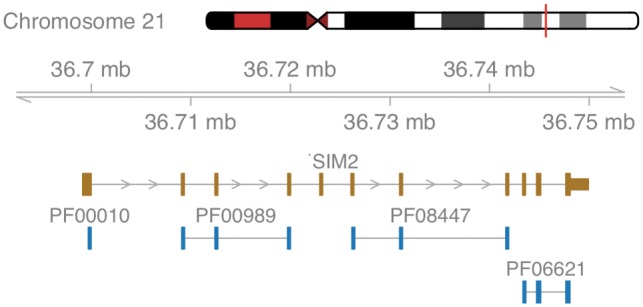
Schematic overview of the gene *SIM2.* Shown are the exons of the gene in brown and all co-locating Pfam protein domains in blue

The first example illustrates filtered data retrieval using *ensembldb* in the context of Down syndrome, a genetic disorder characterized by the presence of all or parts of a third copy of chromosome 21. In our example, we are interested in transcription factors encoded on Chromosome 21 with a basic helix-loop-helix DNA-binding domain, as described by Pfam ID PF00010: given a variable edb of type EnsDb, the simple command gene(edb, filter = ∼ protein_domain_id == “PF00010” & seq_name == “21”) returns the genomic annotations for three genes: *SIM2*, a master regulator of neurogenesis thought to contribute to some phenotypes of Down syndrome (Gardiner and Costa, 2006), and the two genes *OLIG1* and *OLIG2*, triplication of which was shown to cause developmental brain defects (Chakrabarti *et al.*, 2010). Visualization of the genomic neighborhood is accomplished by passing the filter to an *ensembldb* function extracting data for plotting using *Gviz*, as shown in Figure 1.

Another hallmark of *ensembldb* is its capability to convert any position within a protein, transcript or the genome to any other of these three entities, extending the genome to transcript mapping functionality of *GenomicFeatures*. For example, one of the known variants responsible for human red hair color is located at position 16:89920138 (dbSNP ID rs1805009) on the human genome (version GRCh38) and is readily converted by *ensembldb* to position 294 on the respective protein given by Ensembl ID ENSP00000451605 using the command genomeToProtein(GRanges(“16”, IRanges(89920138, width = 1)), edb) with edb as defined in the first example. We furthermore find that this protein is encoded by the *MC1R* gene issuing the *AnnotationDbi*-compatible query command select(edb, keys = “ENSP00000451605”, keytype = “PROTEINID”, columns = “SYMBOL”).

Our annotation packages also contain protein sequence information. Thus, with the call proteins(edb, filter = ∼ protein_id == “ENSP00000451605”)$protein_sequence, we get the protein sequence for the selected ID to find on position 294 an aspartic acid (‘D’), which is in agreement with the reference amino acid of variant Asp294His (Valverde *et al.*, 1995) described by the dbSNP ID cited above.

Expanded code with descriptions and results for these two examples is provided as Supplementary Material and as a Bioconductor package vignette.

Finally, by providing gene annotations and positional information of exons on the genome and supporting the standard Bioconductor interfaces for data retrieval, *ensembldb* can be easily integrated into genome analysis pipelines. An extended example is given in the Supplementary Material, where we present a modified version of the standard Bioconductor RNA-seq workflow (Love *et al.*, 2015).

## 4 Conclusion

Here we have described the Bioconductor package *ensembldb*, which utilizes annotation resources from Ensembl and integrates them into Bioconductor. The separation of source code and annotation data facilitates reproducible research by allowing *ensembldb* to access any set of annotations published in the past. With an extensive filtering system, searches can be customized to meet very specific requirements and powerful coordinate mapping functions enable conversion of coordinates between proteins, transcripts, and the genome. Providing protein and protein domain annotations along with genome-centered annotations makes *ensembldb* also an asset for any post-genome data analysis that aims to combine data from these various domains.

For each new Ensembl release, we create EnsDb annotation databases for all Ensembl vertebrates and plan to provide future continuous support for them via *AnnotationHub*.

*Conflict of Interest*: none declared.

## Supplementary Material

btz031_Supplementary_Information
