# Supplementary material for: ensembldb: an R package to create and use Ensembl-based annotation resources
Source: Bioinformatics. 2019 Jan 25;35(17):3151–3. doi: 10.1093/bioinformatics/btz031 (PMC6736197; doi:10.1093/bioinformatics/btz031)
Supplement: btz031_Supplementary_Information [file btz031_supplementary_information.pdf]

Supplementary data for: *ensembldb: an R package to create and use Ensembl-based annotation resources*

Johannes Rainer, Laurent Gatto and Christian X. Weichenberger

18 December 2018

Contents

1 Query for *helix-loop-helix* transcription factors on chromosome 21 . . . 2

2 Mapping of genomic coordinates to protein-relative positions . . . 7

3 Using `ensembldb` in a standard gene-level RNA-seq workflow . . . 9

3.1 Gene quantification . . . 9

3.2 Differential expression analysis . . . 10

3.3 Annotating results . . . 11

4 Benchmarking the filter framework . . . 13

5 Session information . . . 16

References . . . 17

This documents describes three use cases for *ensembldb*: the first two examples illustrate the filter framework and the coordinate system mapping functionality of `ensembldb`, the latter shows how `ensembldb` annotation resources can be used in an RNA-seq data analysis workflow.

In the last section we compare the performance of queries with and without the filter framework from `ensembdlb`.

1 Query for *helix-loop-helix* transcription factors on chromosome 21

Down syndrome is a genetic disorder characterized by the presence of all or parts of a third copy of chromosome 21. It is associated, among other, with characteristic facial features and mild to moderate intellectual disability. The phenotypes are currently believed to be the result from a gene dosage-dependent increased expression of the genes encoded on chromosome 21 (Lana-Elola et al. 2011). Compared to other gene classes, transcription factors are more likely to have an immediate impact, even due to a moderate over-expression (which might be the result from gene duplication). One of the largest dimerizing transcription factor families is characterized by a *basic helix-loop-helix* domain (Massari and Murre 2000), a protein structural motif facilitating DNA binding.

The example below aims at identifying transcription factors with a basic helix-loop-helix domain (Pfam ID PF00010) that are encoded on chromosome 21. To this end we first load an R-library providing human annotations from Ensembl release 86 and pass the loaded `EnsDb` object along with a filter expression to the `genes` method that retrieves the corresponding genes. Filter expressions have to be written in the form `~ <field> <condition> <value>` with `<field>` representing the database column to be used for the filter. Several such filter expressions can be concatenated with standard R logical expressions (such as `&` or `|`). To get a list of all available filters and their corresponding fields, the `supportedFilters(edb)` function could be used.

```
library(EnsDb.Hsapiens.v86)
edb <- EnsDb.Hsapiens.v86

## Retrieve the genes
gns <- genes(edb, filter = ~ protein_domain_id == "PF00010" & seq_name == "21")
```

The function returned a `GRanges` object with the genomic position of the genes and additional gene-related annotations stored in *metadata* columns.

```
gns
## GRanges object with 3 ranges and 7 metadata columns:
##                   seqnames            ranges strand |         gene_id
##                      <Rle>         <IRanges>  <Rle> |     <character>
##   ENSG00000205927       21 33025845-33029196      + | ENSG00000205927
##   ENSG00000184221       21 33070144-33072420      + | ENSG00000184221
##   ENSG00000159263       21 36699133-36749917      + | ENSG00000159263
##                     gene_name   gene_biotype seq_coord_system      symbol
##                   <character>    <character>      <character> <character>
##   ENSG00000205927       OLIG2 protein_coding       chromosome       OLIG2
##   ENSG00000184221       OLIG1 protein_coding       chromosome       OLIG1
```

```
##   ENSG00000159263        SIM2 protein_coding       chromosome        SIM2
##                     entrezid protein_domain_id
##                       <list>       <character>
##   ENSG00000205927     10215           PF00010
##   ENSG00000184221    116448           PF00010
##   ENSG00000159263      6493           PF00010
##   -------
##   seqinfo: 1 sequence from GRCh38 genome
```

Three transcription factors with a helix-loop-helix domain are encoded on chromosome 21: *SIM2*, which is a master regulator of neurogenesis and is thought to contribute to some specific phenotypes of Down syndrome (Gardiner and Costa 2006) and the two genes *OLIG1* and *OLIG2* for which genetic triplication was shown to cause developmental brain defects (Chakrabarti et al. 2010). To visualize the exonic regions encoding the helix-loop-helix domain of these genes we next retrieve their transcript models and the positions of all Pfam protein domains within the amino acid sequences encoded by these transcripts. We process *SIM2* separately from *OLIG1* and *OLIG2* because the latter are encoded in a narrow region on chromosome 21 and can thus be visualized easily within the same plot. We extract the transcript models for *OLIG1* and *OLIG2* that encode the protein domain using the `getGeneRegionTrackForGviz` function which returns the data in a format that can be directly passed to functions from the `Gviz` Bioconductor package (Hahne and Ivanek 2016) for plotting. Since `Gviz` expects UCSC-style chromosome names instead of the Ensembl chromosome names (e.g. `chr21` instead of `21`), we change the format in which chromosome names are returned by `ensembldb` with the `seqlevelsStyle` method. All subsequent queries to the `EnsDb` database will return chromosome names in UCSC format.

```
## Change chromosome naming style to UCSC
seqlevelsStyle(edb) <- "UCSC"

## Retrieve the transcript models for OLIG1 and OLIG2 that encode the
## the protein domain
txs <- getGeneRegionTrackForGviz(
    edb, filter = ~ genename %in% c("OLIG1", "OLIG2") &
             protein_domain_id == "PF00010")
```

Next we fetch the coordinates of all Pfam protein domains encoded by these transcripts with the `proteins` method, asking for columns `"prot_dom_start"`, `"prot_dom_end"` and `"protein_domain_id"` to be returned by the function. Note that we restrict the results in addition to protein domains defined in Pfam by using an additional filter.

```
pdoms <- proteins(edb, filter = ~ tx_id %in% txs$transcript &
                           protein_domain_source == "pfam",
                  columns = c("protein_domain_id", "prot_dom_start",
                              "prot_dom_end"))
pdoms
## DataFrame with 3 rows and 6 columns
##   protein_domain_id prot_dom_start prot_dom_end      protein_id
##         <character>      <integer>    <integer>     <character>
## 1           PF00010            107          164 ENSP00000371785
## 2           PF00010            110          162 ENSP00000371794
## 3           PF00010            110          162 ENSP00000331040
```

```
##            tx_id protein_domain_source
##      <character>           <character>
## 1 ENST00000382348                  pfam
## 2 ENST00000382357                  pfam
## 3 ENST00000333337                  pfam
```

We next map these protein-relative positions to the genome. We define first an `IRanges` object with the coordinates and submit this to the `proteinToGenome` function for mapping. Besides coordinates, the function requires also the respective protein identifiers which we supply as names.

```
pdoms_rng <- IRanges(start = pdoms$prot_dom_start, end = pdoms$prot_dom_end,
                     names = pdoms$protein_id)

pdoms_gnm <- proteinToGenome(pdoms_rng, edb)
```

The result is a `list` of `GRanges` objects with the genomic coordinates at which the protein domains are encoded, one for each of the input protein domains. Additional information such as the protein ID, the encoding transcript and the exons of the respective transcript in which the domain is encoded are provided as metadata columns.

```
pdoms_gnm
## $ENSP00000371785
## GRanges object with 1 range and 7 metadata columns:
##       seqnames            ranges strand |      protein_id           tx_id
##          <Rle>         <IRanges>  <Rle> |     <character>     <character>
##   [1]    chr21 33070565-33070738      + | ENSP00000371785 ENST00000382348
##                exon_id exon_rank    cds_ok protein_start protein_end
##            <character> <integer> <logical>     <integer>   <integer>
##   [1] ENSE00001491811         1      TRUE           107         164
##   -------
##   seqinfo: 1 sequence from GRCh38 genome
##
## $ENSP00000371794
## GRanges object with 1 range and 7 metadata columns:
##       seqnames            ranges strand |      protein_id           tx_id
##          <Rle>         <IRanges>  <Rle> |     <character>     <character>
##   [1]    chr21 33027190-33027348      + | ENSP00000371794 ENST00000382357
##                exon_id exon_rank    cds_ok protein_start protein_end
##            <character> <integer> <logical>     <integer>   <integer>
##   [1] ENSE00001491833         2      TRUE           110         162
##   -------
##   seqinfo: 1 sequence from GRCh38 genome
##
## $ENSP00000331040
## GRanges object with 1 range and 7 metadata columns:
##       seqnames            ranges strand |      protein_id           tx_id
##          <Rle>         <IRanges>  <Rle> |     <character>     <character>
##   [1]    chr21 33027190-33027348      + | ENSP00000331040 ENST00000333337
##                exon_id exon_rank    cds_ok protein_start protein_end
##            <character> <integer> <logical>     <integer>   <integer>
```

```
##   [1] ENSE00001379164         1      TRUE           110         162
##   -------
##   seqinfo: 1 sequence from GRCh38 genome
```

Column `cds_ok` in the result object indicates whether the length of the CDS of the encoding transcript matches the length of the protein sequence. For transcripts with unknown 3' and/or 5' CDS ends these will differ. The mapping result has to be re-organized before being plotted: `Gviz` expects a single `GRanges` object, with specific metadata columns for the grouping of the individual genomic regions. This is performed in the code block below.

```
## Convert the list to a GRanges with grouping information
pdoms_gnm_grng <- unlist(GRangesList(pdoms_gnm))
pdoms_gnm_grng$id <- rep(pdoms$protein_domain_id, lengths(pdoms_gnm))
pdoms_gnm_grng$grp <- rep(1:nrow(pdoms), lengths(pdoms_gnm))

pdoms_gnm_grng
## GRanges object with 3 ranges and 9 metadata columns:
##                    seqnames            ranges strand |      protein_id
##                       <Rle>         <IRanges>  <Rle> |     <character>
##   ENSP00000371785     chr21 33070565-33070738      + | ENSP00000371785
##   ENSP00000371794     chr21 33027190-33027348      + | ENSP00000371794
##   ENSP00000331040     chr21 33027190-33027348      + | ENSP00000331040
##                             tx_id         exon_id exon_rank    cds_ok
##                       <character>     <character> <integer> <logical>
##   ENSP00000371785 ENST00000382348 ENSE00001491811         1      TRUE
##   ENSP00000371794 ENST00000382357 ENSE00001491833         2      TRUE
##   ENSP00000331040 ENST00000333337 ENSE00001379164         1      TRUE
##                   protein_start protein_end          id       grp
##                       <integer>   <integer> <character> <integer>
##   ENSP00000371785           107         164     PF00010         1
##   ENSP00000371794           110         162     PF00010         2
##   ENSP00000331040           110         162     PF00010         3
##   -------
##   seqinfo: 1 sequence from GRCh38 genome
```

We next define the individual tracks we want to visualize and plot them with the `plotTracks` function from the `Gviz` package.

```
library(Gviz)

## Define the individual tracks:
## - Ideagram
ideo_track <- IdeogramTrack(genome = "hg38", chromosome = "chr21")
## - Genome axis
gaxis_track <- GenomeAxisTrack()
## - Transcripts
gene_track <- GeneRegionTrack(txs, showId = TRUE, just.group = "right",
                              name = "", geneSymbol = TRUE, size = 0.5)
## - Protein domains
pdom_track <- AnnotationTrack(pdoms_gnm_grng, group = pdoms_gnm_grng$grp,
                              id = pdoms_gnm_grng$id, groupAnnotation = "id",
```

```
                                        just.group = "right", shape = "box",
                                        name = "Protein domains", size = 0.5)

## Generate the plot
plotTracks(list(ideo_track, gaxis_track, gene_track, pdom_track))
```

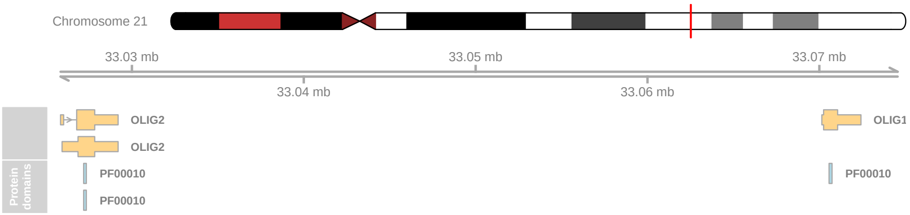

Figure 1: **Transcripts of genes OLIG1 and OLIG2 encoding a helix-loop-helix protein domain**
Shown are all transcripts of the genes OLIG1 and OLIG2 that encode a protein with a helix-loop-helix protein domain (PF00010). Genomic positions encoding protein domains defined in Pfam are shown in light blue.

All transcripts are relatively short with the full coding region being in a single exon. Also, both transcripts encode a protein with a single protein domain, the helix-loop-helix domain PF00010.

Next we repeat the analysis for *SIM2* by first fetching all of its transcript variants encoding the PF00010 Pfam protein domain from the database. Subsequently we retrieve all Pfam protein domains encoded in these transcripts.

```
## Fetch all SIM2 transcripts encoding PF00010
txs <- getGeneRegionTrackForGviz(edb, filter = ~ genename == "SIM2" &
                                          protein_domain_id == "PF00010")
## Fetch all Pfam protein domains within these transcripts
pdoms <- proteins(edb, filter = ~ tx_id %in% txs$transcript &
                              protein_domain_source == "pfam",
                      columns = c("protein_domain_id", "prot_dom_start",
                                  "prot_dom_end"))
```

At last we have to map the protein domain coordinates to the genome and prepare the data for the plot. Since the code is essentially identical to the one for *OLIG1* and *OLIG2* it is not displayed.

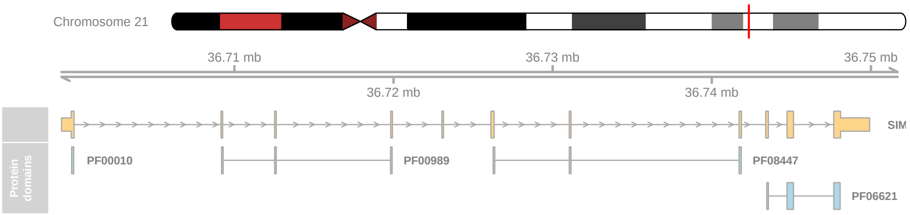

Figure 2: **Transcripts of the gene SIM2 encoding a helix-loop-helix domain**
Shown are all transcripts of SIM2 encoding a protein with a helix-loop-helix protein domain (PF00010). Genomic positions encoding protein domains defined in Pfam are shown in light blue.

The *SIM2* transcript encodes a protein with in total 4 protein domains. The helix-loop-helix domain PF00010 is encoded in its first exon.

2 Mapping of genomic coordinates to protein-relative positions

One of the known mutations for human red hair color is located at position 16:89920138 (dbSNP ID rs1805009) on the human genome (version GRCh38). Below we map this genomic coordinate to the respective coordinate within the protein sequence encoded at that location using the `genomeToProtein` function. Note that we use `"chr16"` as the name of the chromosome, since we changed the chromosome naming style to UCSC in the previous example.

```
gnm_pos <- GRanges("chr16", IRanges(89920138, width = 1))
prt_pos <- genomeToProtein(gnm_pos, edb)
prt_pos
## IRangesList of length 1
## [[1]]
## IRanges object with 3 ranges and 8 metadata columns:
##                      start       end     width |           tx_id    cds_ok
##                  <integer> <integer> <integer> |     <character> <logical>
##   ENSP00000451605       294       294         1 | ENST00000555147      TRUE
##   ENSP00000451760       294       294         1 | ENST00000555427      TRUE
##   ENSP00000451560       294       294         1 | ENST00000556922      TRUE
##                          exon_id exon_rank seq_start   seq_end    seq_name
##                      <character> <integer> <integer> <integer> <character>
##   ENSP00000451605 ENSE00002458332         1  89920138  89920138       chr16
##   ENSP00000451760 ENSE00002477640         3  89920138  89920138       chr16
##   ENSP00000451560 ENSE00002507842         1  89920138  89920138       chr16
##                    seq_strand
##                  <character>
##   ENSP00000451605           *
##   ENSP00000451760           *
##   ENSP00000451560           *
```

The genomic position could thus be mapped to the amino acid 294 in each of the 3 proteins listed above. Using the `select` function we retrieve the HGNC symbol of the gene for these 3 proteins.

```
select(edb, keys = ~ protein_id == names(prt_pos[[1]]), columns = "SYMBOL")
##          SYMBOL       PROTEINID
## 1 RP11-566K11.2 ENSP00000451560
## 2          MC1R ENSP00000451605
## 3          MC1R ENSP00000451760
```

Two proteins are from the *MC1R* gene and one from *RP11-566K11.2* (ENSG00000198211) a gene which exons overlap exons from *MC1R* as well as exons of the more downstream located gene *TUBB3*. To visualize this we first fetch transcripts overlapping the genomic position of interest and subsequently all additional transcripts within the region defined by the most downstream and upstream exons of the transcripts.

```
## Get transcripts overlapping the genomic position.
txs <- getGeneRegionTrackForGviz(edb, filter = GRangesFilter(gnm_pos))
```

```
## Get all transcripts within the region from the start of the most 5'
## and end of the most 3' exon.
all_txs <- getGeneRegionTrackForGviz(
    edb, filter = GRangesFilter(range(txs), type = "within"))

## Plot the data
## - Ideogram
ideo_track <- IdeogramTrack(genome = "hg38", chromosome = "chr16")
## - Genome axis
gaxis_track <- GenomeAxisTrack()
## - Transcripts
gene_track <- GeneRegionTrack(all_txs, showId = TRUE, just.group = "right",
                              name = "", geneSymbol = TRUE, size = 0.5)
## - highlight the region.
hl_track <- HighlightTrack(list(gaxis_track, gene_track), range = gnm_pos)

## Generate the plot
plotTracks(list(ideo_track, hl_track))
```

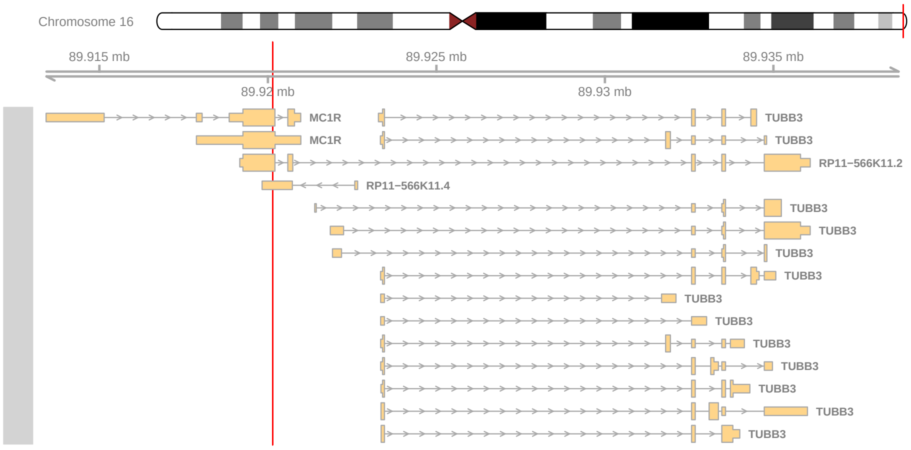

Figure 3: Transcripts overlapping, or close to, the genomic position of interest
Shown are all transcripts. The genomic position of the variant is highlighted in red.

The plot above visualizes the expanded genomic region of the variant (indicated with a vertical red line) including all transcripts (from both strands) encoded in the region. In total 4 genes are present in the region: *MC1R*, the *MC1R-TUBB3* read-through gene *RP11-566K11.2*, the non-coding gene *RP11-566K11.4* located on the reverse strand and *TUBB3*. Exons of transcripts from the former 3 genes overlap the genomic position of the variant. Using the `proteins` method we next extract the sequences of the proteins encoded by the 3 transcripts (two of *MC1R* and one of *RP11-566K11.2*) and determine the amino acid at position 294 in these. To retrieve the results in a format most suitable for the representation of amino acid sequences we specify `return.type = "AAStringSet"` in the `proteins` call.

```
## Get the amino acid sequences for the 3 transcripts
prt_seq <- proteins(edb, return.type = "AAStringSet",
                    filter = ~ protein_id == names(prt_pos[[1]]))
## Extract the amino acid at position 294
subseq(prt_seq, start = 294, end = 294)
```

```
##   A AAStringSet instance of length 3
##     width seq                                              names
## [1]     1 D                                                ENSP00000451560
## [2]     1 D                                                ENSP00000451605
## [3]     1 D                                                ENSP00000451760
```

The amino acid at position 294 is for all an aspartic acid ("D") which is in agreement with the reference amino acid of mutation Asp294His (Valverde et al. 1995) described by the dbSNP ID of this example.

3 Using `ensembldb` in a standard gene-level RNA-seq workflow

This workflow is based on the RNA-seq workflow (Love et al. 2015) and shows how the `ensembldb` package can be used for all annotation-related tasks in that analysis. In brief, the workflow describes the analysis of an RNA-seq data set after alignment of the reads to the genome with the aim to identify genes that are differentially expressed in airway smooth muscle cells after treatment with the synthetic glucocorticoid dexamethasone, a drug with anti-inflammatory effects. Glucocorticoids are used, for example, by people with asthma to reduce inflammation of the airways. Note that here we focus only on the parts of the analysis in which `ensembldb` functionality and data are used, i.e. the gene quantification step and the annotation of the analysis results. Other topics from the workflow such as the visualization and exploratory data analysis are not covered here. Also, please refer to the original workflow for background information and detailed descriptions of the methodology used.

Below we load the `airway` data package that provides all required files for the present analysis (including BAM files with a subset of the alignment results of the raw reads to the genome GRCh37) and load a text file with sample descriptions from this package.

```
library(airway)
sampleTable <- read.csv(system.file("extdata/sample_table.csv",
                                    package = "airway"),
                        row.names = 1)
```

3.1 Gene quantification

After alignment of the reads to the genome, gene abundance estimates can be generated by counting the number of reads falling within the exon boundaries of a gene. The BAM files with the alignment results of the present data set are provided by the `airway` package. Below we load these into a `BamFileList` object defined in the *Rsamtools* package which enables reading alignment information from BAM files.

```
library(Rsamtools)
bamfiles <- BamFileList(paste0(system.file("extdata", package = "airway"),
                               "/", sampleTable$Run, "_subset.bam"),
                        yieldSize = 2000000)
```

For gene quantification we use the `summarizeOverlaps` function from the *GenomicAlignments* package (Lawrence et al. 2013) that requires, apart from the aligned reads, also the genomic positions of all exons of all genes. In contrast to the original workflow, we retrieve these directly from the appropriate *ensembldb* `EnsDb` database that we load in the code block below. Since the reads in this RNA-seq data set were aligned against GRCh37, we load an `ensembldb` resource for an Ensembl release matching that genome version (i.e. release 75). The genomic positions of all exons grouped by gene are extracted from the database with the `exonsBy` function.

```
library(ensembldb)
library(EnsDb.Hsapiens.v75)
ebg <- exonsBy(EnsDb.Hsapiens.v75, "gene")
```

With this we can now proceed to the read counting with the `summarizeOverlaps` function using the same settings as in the original workflow.

```
library(GenomicAlignments)
se <- summarizeOverlaps(features = ebg, reads = bamfiles,
                        mode = "Union", singleEnd = FALSE,
                        ignore.strand = TRUE, fragments = TRUE)
```

At last we add the sample metadata to the result object (a `SummarizedExperiment`) and ensure that `untrt` is the reference level for the variable specifying the treatment of the samples (i.e. `dex`).

```
## Add sample metadata
colData(se) <- DataFrame(sampleTable)
se$dex <- relevel(se$dex, "untrt")
```

3.2 Differential expression analysis

We use *DESeq2* (Love, Huber, and Anders 2014) for the differential expression analysis to identify genes that are deregulated upon treatment with the synthetic glucocorticoid dexamethasone (dex). The `SumarizedExperiment` with the gene quantifications generated in the previous section contains only reads aligned against chromosome 1. Thus, as in the original workflow, we load a prepared `SummarizedExperiment` containing read counts for genes from all chromosomes (which is also provided in the `airway` package).

```
data("airway")
se <- airway
se$dex <- relevel(se$dex, "untrt")
```

We next build a `DESeqDataSet` from this object also specifying the experimental layout with the `design` argument.

```
library(DESeq2)
dds <- DESeqDataSet(se, design = ~ cell + dex)
```

Before proceeding to the differential expression analysis we remove rows (genes) with only zeros or very low read counts.

```
dds <- dds[rowSums(counts(dds)) > 1, ]
```

As we have already specified an experimental design when creating the `DESeqDataSet`, we can run the differential expression analysis on the raw counts with a single call to the function `DESeq`:

```
dds <- DESeq(dds)
## estimating size factors
## estimating dispersions
## gene-wise dispersion estimates
## mean-dispersion relationship
## final dispersion estimates
## fitting model and testing
```

We can now extract the estimated log2 fold changes and p values for the last variable in the design formula.

```
res <- results(dds)
head(res[order(res$pvalue), ])
## log2 fold change (MLE): dex trt vs untrt
## Wald test p-value: dex trt vs untrt
## DataFrame with 6 rows and 6 columns
##                         baseMean   log2FoldChange             lfcSE
##                        <numeric>        <numeric>         <numeric>
## ENSG00000152583 997.439773207048 4.57491904614571 0.184056290144421
## ENSG00000165995 495.092906698546 3.29106191054141  0.13317371150066
## ...                          ...              ...               ...
## ENSG00000189221 2341.76725275591  3.3535801702967 0.141782454259944
## ENSG00000211445 12285.6151498691 3.73040303801649 0.165830587883941
##                             stat                pvalue                  padj
##                        <numeric>             <numeric>             <numeric>
## ENSG00000152583 24.8560863774662 2.22231970363871e-136 3.99884207472749e-132
## ENSG00000165995 24.7125492971269 7.83975707156014e-135 7.05342943728266e-131
## ...                          ...                   ...                   ...
## ENSG00000189221 23.6529984461141 1.09937071872851e-123 3.95641534256016e-120
## ENSG00000211445 22.4952651113272 4.61813770361409e-112 1.38497949731387e-108
```

3.3 Annotating results

As a last step we want to annotate our result table. In contrast to the original workflow, we extract annotations for the Ensembl gene identifiers from the `EnsDb` database we have already used in the gene quantification step above. This simplifies the analysis and additionally ensures that both annotations as well as positional information used for the read counting are from the same data release.

Below we use `ensembldb`'s `genes` function to extract all gene annotations available in the `EnsDb` database and return the results as a `DataFrame`.

```
anns <- genes(EnsDb.Hsapiens.v75, return.type = "DataFrame")
anns
## DataFrame with 64102 rows and 10 columns
```

```
##               gene_id   gene_name gene_biotype gene_seq_start gene_seq_end
##           <character> <character>  <character>      <integer>    <integer>
## 1     ENSG00000223972     DDX11L1   pseudogene          11869        14412
## 2     ENSG00000227232      WASH7P   pseudogene          14363        29806
## ...               ...         ...          ...            ...          ...
## 64101 ENSG00000231514     FAM58CP   pseudogene       28772667     28773306
## 64102 ENSG00000235857     CTBP2P1   pseudogene       59001391     59001635
##          seq_name seq_strand seq_coord_system      symbol
##       <character>  <integer>      <character> <character>
## 1               1          1       chromosome     DDX11L1
## 2               1         -1       chromosome      WASH7P
## ...           ...        ...              ...         ...
## 64101           Y         -1       chromosome     FAM58CP
## 64102           Y          1       chromosome     CTBP2P1
##                      entrezid
##                        <list>
## 1     c(100287596, 100287102)
## 2        c(100287171, 653635)
## ...                       ...
## 64101                      NA
## 64102                      NA
```

Annotations include the positional information of the genes, their name/symbol, their biotype and all NCBI Entrez gene identifiers associated with them. The mapping from Ensembl gene IDs to Entrez gene IDs can be one to many. We thus collapse multiple Entrez gene IDs for one Ensembl gene into a comma separated string.

```
anns$entrezid <- vapply(anns$entrezid,
                        function(z) {
                            if (all(is.na(z)))
                                NA_character_
                            else paste(z, collapse = ",")
                        }, character(1))
anns
## DataFrame with 64102 rows and 10 columns
##               gene_id   gene_name gene_biotype gene_seq_start gene_seq_end
##           <character> <character>  <character>      <integer>    <integer>
## 1     ENSG00000223972     DDX11L1   pseudogene          11869        14412
## 2     ENSG00000227232      WASH7P   pseudogene          14363        29806
## ...               ...         ...          ...            ...          ...
## 64101 ENSG00000231514     FAM58CP   pseudogene       28772667     28773306
## 64102 ENSG00000235857     CTBP2P1   pseudogene       59001391     59001635
##          seq_name seq_strand seq_coord_system      symbol
##       <character>  <integer>      <character> <character>
## 1               1          1       chromosome     DDX11L1
## 2               1         -1       chromosome      WASH7P
## ...           ...        ...              ...         ...
## 64101           Y         -1       chromosome     FAM58CP
## 64102           Y          1       chromosome     CTBP2P1
##                entrezid
##             <character>
```

```
## 1     100287596,100287102
## 2        100287171,653635
## ...                   ...
## 64101                  NA
## 64102                  NA
```

At last we combine the results and annotation tables based on the Ensembl gene identifier.

```
rownames(anns) <- anns$gene_id
res <- cbind(anns[rownames(res), ], res)
head(res[order(res$pvalue), c("gene_name", "log2FoldChange", "padj")])
## DataFrame with 6 rows and 3 columns
##                   gene_name   log2FoldChange                  padj
##                 <character>        <numeric>             <numeric>
## ENSG00000152583     SPARCL1 4.57491904614571 3.99884207472749e-132
## ENSG00000165995      CACNB2 3.29106191054141 7.05342943728266e-131
## ...                     ...              ...                   ...
## ENSG00000189221        MAOA  3.3535801702967 3.95641534256016e-120
## ENSG00000211445        GPX3 3.73040303801649 1.38497949731387e-108
```

4 Benchmarking the filter framework

Filters in `ensembldb` are *translated* into the underlying SQL commands which leads to a significant performance gain on data retrieval if only subsets of the data are fetched. In this section we compare the performances of data retrieval in the *classical* way, i.e. by first extracting the full data from the database with subsequent sub-setting in R, and data retrieval using the `ensembldb` filter framework.

```
library(EnsDb.Hsapiens.v86)
library(microbenchmark)
edb <- EnsDb.Hsapiens.v86
```

The first use case is to retrieve all gene-related annotations for the *SIM2* gene from Section 1. The classical approach is to get all gene annotations and subset the results to those for which the gene name matches *SIM2*.

```
gns <- genes(edb)
gns[gns$gene_name == "SIM2", ]
## GRanges object with 1 range and 6 metadata columns:
##                   seqnames            ranges strand |         gene_id
##                      <Rle>         <IRanges>  <Rle> |     <character>
##   ENSG00000159263       21 36699133-36749917      + | ENSG00000159263
##                     gene_name   gene_biotype seq_coord_system      symbol
##                   <character>    <character>      <character> <character>
##   ENSG00000159263        SIM2 protein_coding       chromosome        SIM2
##                   entrezid
##                     <list>
##   ENSG00000159263     6493
##   -------
##   seqinfo: 357 sequences from GRCh38 genome
```

Using the filter framework this is simplified to:

```
genes(edb, filter = ~ gene_name == "SIM2")
## GRanges object with 1 range and 6 metadata columns:
##                     seqnames            ranges strand |         gene_id
##                        <Rle>         <IRanges>  <Rle> |     <character>
##   ENSG00000159263       21 36699133-36749917      + | ENSG00000159263
##                     gene_name   gene_biotype seq_coord_system      symbol
##                   <character>    <character>      <character> <character>
##   ENSG00000159263        SIM2 protein_coding       chromosome        SIM2
##                     entrezid
##                       <list>
##   ENSG00000159263     6493
##   -------
##   seqinfo: 1 sequence from GRCh38 genome
```

Next we compare the performance of both approaches using the `microbenchmark` function evaluating each expression 8 times. The output from the benchmark with a summary of the evaluation times is displayed below. The calculations were performed on a MacBook Pro (15-inch 2016) with a quad-core 2.9 GHz Intel Core i7 CPU and 16 GB memory.

```
microbenchmark(
{
    gns <- genes(edb)
    gns[gns$gene_name == "SIM2"]
},
genes(edb, filter = ~ gene_name == "SIM2"), times = 8)
## Unit: milliseconds
##                                                          expr       min
##  {     gns <- genes(edb)     gns[gns$gene_name == "SIM2"] } 956.41633
##                     genes(edb, filter = ~gene_name == "SIM2")  15.27729
##         lq       mean     median         uq        max neval cld
##  969.91094 1025.96638 1028.14788 1070.02981 1115.13742     8   b
##   16.26708   25.52677   18.01566   19.27636   81.81865     8  a
```

We see a clear performance advantage of the filter framework. With the classical approach it took on average 1 second to extract the relevant annotation, while filtering the data with the filter framework reduced this to less than 20 milliseconds which represents an about 60-fold performance increase.

In the next example we want to get the exon positions, grouped by gene, for genes that are encoded on chromosome X. First we use again the classical approach fetching all information and sub-setting later.

```
exns <- exonsBy(edb, "gene")
exns[seqnames(exns) == "X"]
## GRangesList object of length 63970:
## $ENSG00000000003
## GRanges object with 20 ranges and 1 metadata column:
##        seqnames              ranges strand |         exon_id
##           <Rle>           <IRanges>  <Rle> |     <character>
```

```
##    [1]        X 100639945-100639991      - | ENSE00001828996
##    [2]        X 100636793-100637104      - | ENSE00001863395
##    ...      ...                 ...    ... .             ...
##   [19]        X 100628670-100629986      - | ENSE00001459322
##   [20]        X 100627109-100629986      - | ENSE00003730948
##
## ...
## <63969 more elements>
## -------
## seqinfo: 357 sequences from GRCh38 genome
```

With the filter framework the task is simplified to

```
exonsBy(edb, "gene", filter = ~ seq_name == "X")
## GRangesList object of length 2399:
## $ENSG00000000003
## GRanges object with 20 ranges and 1 metadata column:
##        seqnames              ranges strand |         exon_id
##           <Rle>           <IRanges>  <Rle> |     <character>
##    [1]        X 100639945-100639991      - | ENSE00001828996
##    [2]        X 100636793-100637104      - | ENSE00001863395
##    ...      ...                 ...    ... .             ...
##   [19]        X 100628670-100629986      - | ENSE00001459322
##   [20]        X 100627109-100629986      - | ENSE00003730948
##
## ...
## <2398 more elements>
## -------
## seqinfo: 1 sequence from GRCh38 genome
```

Next we compare the performance of both approaches.

```
microbenchmark(
{
    exns <- exonsBy(edb, "gene")
    exns[seqnames(exns) == "X"]
},
exonsBy(edb, "gene", filter = ~ seq_name == "X"), times = 8)
## Unit: milliseconds
##                                                                  expr
##  {     exns <- exonsBy(edb, "gene")     exns[seqnames(exns) == "X"] }
##                       exonsBy(edb, "gene", filter = ~seq_name == "X")
##         min         lq      mean     median         uq        max neval cld
##  13229.8597 13334.8555 13578.835 13614.2404 13781.5302 13939.5648     8   b
##    251.6005   256.8368   266.176   262.2042   271.1462   297.4331     8  a
```

The performance gain when using the filter framework was again remarkable (on average about 13 seconds with the classical approach compared to approximately 0.25 seconds with the filter framework).

Summarizing, filters in `ensembldb` allow to retrieve only subsets of the full annotations available in `EnsDb` databases and their use leads to a significant performance gain in such cases. In addition, the filtering framework simplifies the sub-setting workflow considerably and provides a clearer syntax.

5 Session information

```
sessionInfo()
## R version 3.5.1 (2018-07-02)
## Platform: x86_64-apple-darwin18.2.0/x86_64 (64-bit)
## Running under: macOS  10.14.3
##
## Matrix products: default
## BLAS: /System/Library/Frameworks/Accelerate.framework/Versions/A/Frameworks/vecLib.framework/Versions/A/li
## LAPACK: /System/Library/Frameworks/Accelerate.framework/Versions/A/Frameworks/vecLib.framework/Versions/A/
##
## locale:
## [1] en_US.UTF-8/en_US.UTF-8/en_US.UTF-8/C/en_US.UTF-8/en_US.UTF-8
##
## attached base packages:
##  [1] grid      stats4    parallel  stats     graphics  grDevices utils
##  [8] datasets  methods   base
##
## other attached packages:
##  [1] microbenchmark_1.4-6        DESeq2_1.22.1
##  [3] GenomicAlignments_1.18.0    EnsDb.Hsapiens.v75_2.99.0
##  [5] Rsamtools_1.34.0            Biostrings_2.50.1
##  [7] XVector_0.22.0              airway_1.2.0
##  [9] SummarizedExperiment_1.12.0 DelayedArray_0.8.0
## [11] BiocParallel_1.16.2         matrixStats_0.54.0
## [13] Gviz_1.26.3                 EnsDb.Hsapiens.v86_2.99.0
## [15] ensembldb_2.6.3             AnnotationFilter_1.6.0
## [17] GenomicFeatures_1.34.1      AnnotationDbi_1.44.0
## [19] Biobase_2.42.0              GenomicRanges_1.34.0
## [21] GenomeInfoDb_1.18.1         IRanges_2.16.0
## [23] S4Vectors_0.20.1            BiocGenerics_0.28.0
## [25] BiocStyle_2.10.0            rmarkdown_1.11
##
## loaded via a namespace (and not attached):
##  [1] TH.data_1.0-9            colorspace_1.3-2
##  [3] biovizBase_1.30.0        htmlTable_1.12
##  [5] base64enc_0.1-3          dichromat_2.0-0
##  [7] rstudioapi_0.8           bit64_0.9-7
##  [9] mvtnorm_1.0-8            codetools_0.2-15
## [11] splines_3.5.1            geneplotter_1.60.0
## [13] knitr_1.21               Formula_1.2-3
## [15] annotate_1.60.0          cluster_2.0.7-1
## [17] BiocManager_1.30.4       compiler_3.5.1
## [19] httr_1.4.0               backports_1.1.2
```

```
## [21] assertthat_0.2.0         Matrix_1.2-15
## [23] lazyeval_0.2.1           acepack_1.4.1
## [25] htmltools_0.3.6          prettyunits_1.0.2
## [27] tools_3.5.1              bindrcpp_0.2.2
## [29] gtable_0.2.0             glue_1.3.0
## [31] GenomeInfoDbData_1.2.0   dplyr_0.7.8
## [33] tinytex_0.9              Rcpp_1.0.0
## [35] rtracklayer_1.42.1       xfun_0.4
## [37] stringr_1.3.1            XML_3.98-1.16
## [39] zlibbioc_1.28.0          MASS_7.3-51.1
## [41] zoo_1.8-4                scales_1.0.0
## [43] BSgenome_1.50.0          VariantAnnotation_1.28.3
## [45] hms_0.4.2                ProtGenerics_1.14.0
## [47] sandwich_2.5-0           RColorBrewer_1.1-2
## [49] yaml_2.2.0               curl_3.2
## [51] memoise_1.1.0            gridExtra_2.3
## [53] ggplot2_3.1.0            biomaRt_2.38.0
## [55] rpart_4.1-13             latticeExtra_0.6-28
## [57] stringi_1.2.4            RSQLite_2.1.1
## [59] genefilter_1.64.0        checkmate_1.8.5
## [61] rlang_0.3.0.1            pkgconfig_2.0.2
## [63] bitops_1.0-6             evaluate_0.12
## [65] lattice_0.20-38          purrr_0.2.5
## [67] bindr_0.1.1              htmlwidgets_1.3
## [69] bit_1.1-14               tidyselect_0.2.5
## [71] plyr_1.8.4               magrittr_1.5
## [73] bookdown_0.8             R6_2.3.0
## [75] Hmisc_4.1-1              multcomp_1.4-8
## [77] DBI_1.0.0                pillar_1.3.0
## [79] foreign_0.8-71           survival_2.43-3
## [81] RCurl_1.95-4.11          nnet_7.3-12
## [83] tibble_1.4.2             crayon_1.3.4
## [85] progress_1.2.0           locfit_1.5-9.1
## [87] data.table_1.11.8        blob_1.1.1
## [89] digest_0.6.18            xtable_1.8-3
## [91] munsell_0.5.0
```

References

Chakrabarti, Lina, Tyler K Best, Nathan P Cramer, Rosalind S E Carney, John T R Isaac, Zygmunt Galdzicki, and Tarik F Haydar. 2010. "Olig1 and Olig2 triplication causes developmental brain defects in Down syndrome." *Nature Neuroscience* 13 (8): 927–34.

Gardiner, Katheleen, and Alberto C S Costa. 2006. "The proteins of human chromosome 21." *American Journal of Medical Genetics. Part C, Seminars in Medical Genetics* 142C (3): 196–205.

Hahne, Florian, and Robert Ivanek. 2016. "Visualizing Genomic Data Using Gviz and Bioconductor." *Methods in Molecular Biology (Clifton, N.J.)* 1418 (Chapter 16): 335–51.

Lana-Elola, Eva, Sheona D Watson-Scales, Elizabeth M C Fisher, and Victor L J Tybulewicz. 2011. "Down syndrome: searching for the genetic culprits." *Disease Models & Mechanisms* 4 (5): 586–95.

Lawrence, Michael, Wolfgang Huber, Hervé Pagès, Patrick Aboyoun, Marc Carlson, Robert Gentleman, Martin T Morgan, and Vincent J Carey. 2013. "Software for computing and annotating genomic ranges." *PLoS Computational Biology* 9 (8): e1003118. https://doi.org/10.1371/journal.pcbi.1003118.

Love, Michael I, Simon Anders, Vladislav Kim, and Wolfgang Huber. 2015. "RNA-Seq workflow: gene-level exploratory analysis and differential expression." *F1000Res.* 4: 1070. https://doi.org/10.12688/f1000research.7035.1.

Love, Michael I, Wolfgang Huber, and Simon Anders. 2014. "Moderated estimation of fold change and dispersion for RNA-seq data with DESeq2." *Genome Biology* 15 (12): 550. https://doi.org/10.1186/s13059-014-0550-8.

Massari, M E, and C Murre. 2000. "Helix-loop-helix proteins: regulators of transcription in eucaryotic organisms." *Molecular and Cellular Biology* 20 (2): 429–40.

Valverde, P, E Healy, I Jackson, J L Rees, and A J Thody. 1995. "Variants of the melanocyte-stimulating hormone receptor gene are associated with red hair and fair skin in humans." *Nature Genetics* 11 (3): 328–30.
